# 100 Hz neutron radiography at the BOA beamline using a parabolic focussing guide

**DOI:** 10.1016/j.mex.2016.10.001

**Published:** 2016-10-06

**Authors:** Pavel Trtik, Manuel Morgano, Roman Bentz, Eberhard Lehmann

**Affiliations:** a Neutron Imaging and Activation Group, Laboratory for Neutron Scattering and Imaging Paul Scherrer Institut, 5232 Villigen PSI, Switzerland; b Laboratory of Radiochemistry and Environmental Chemistry, Paul Scherrer Institut, 5232 Villigen PSI, Switzerland

**Keywords:** Neutron imaging, High temporal resolution neutron imaging, Parabolic focussing guide, Two phase flow, D_2_O, H_2_O, Interaction of H_2_O and D_2_O

## Abstract

The recent developments in scientific complementary metal oxide semiconductor (sCMOS) detector technology allow for imaging of relevant processes with very high temporal resolution with practically negligible readout time. However, it is neutron intensity that limits the high temporal resolution neutron imaging. In order to partially overcome the neutron intensity problem for the high temporal resolution imaging, a parabolic neutron focussing guide was utilized in the test arrangement and placed upstream the detector in such a manner that the focal point of the guide was positioned slightly behind the scintillator screen. In such a test arrangement, the neutron flux can be increased locally by about one order of magnitude, albeit with the reduced spatial resolution due to the increased divergence of the neutron beam. In a pilot test application, an in-situ titration system allowing for a remote delivery of well-defined volumes of liquids onto the sample stage was utilized. The process of droplets of water (H_2_O) falling into the container filled with heavy water (D_2_O) and the subsequent process of the interaction and mixing of the two liquids were imaged with temporal resolution of 0.01 s.

• Combination of neutron focussing device and use of sCMOS detector allows for very high temporal resolution neutron imaging to be achieved (albeit with reduced spatial resolution and field of view).
• In-situ neutron imaging titration device for liquid interaction experiments.
• Interaction of otherwise indiscernible liquids (H_2_O and D_2_O) visualized using neutron radiography with 0.01 s temporal resolution.

## Graphical abstract

**Figure:**
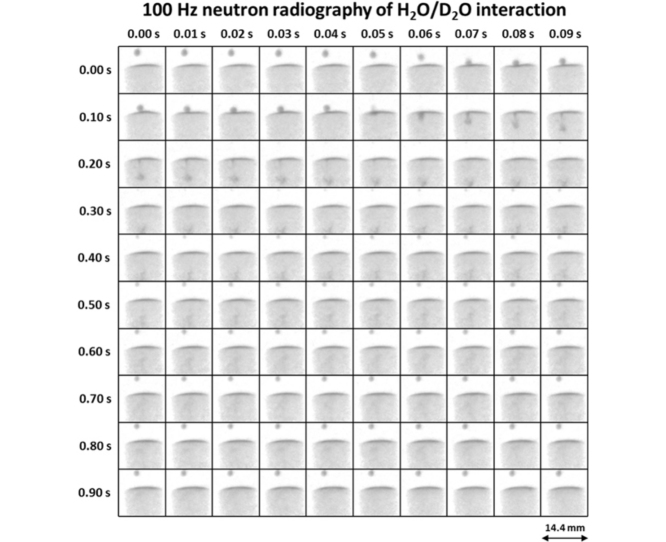

## Introduction

High temporal resolution neutron imaging is a technique from which several domains of science and engineering may profit, as there are large number of processes (e.g. turbulence, two phase flow, mixing, etc.) for which a high temporal resolution neutron imaging is an appropriate technique of investigation.

It is the recent developments in sCMOS detector technology that allow for imaging of such processes with very high temporal resolution. The negligible readout-time and low read-out noise of the sCMOS cameras therefore allow for the “continuous” observation of non-cyclic processes with high-temporal resolution (routinely up to 0.01 s).

The examples of high temporal resolution neutron imaging of industrially relevant samples utilizing the sCMOS technology include: the visualization of flows in liquid metals with temporal resolution of approximately 0.03 s in 2D [1], and on-the-fly tomography of water uptake in roots with sub-minute resolution in 3D [2]. An overview of the high-temporal resolution imaging for studies of porous media has been recently provided by Kaestner et al. [3]. It is necessary to mention here that the neutron imaging of even higher temporal resolution has hitherto been available only for the case of repetitive/cyclic processes (e.g. imaging of running motors) using the stroboscopic modality of the neutron imaging [4].

However, should one like to investigate non-cyclic processes using neutron imaging with 0.01 s temporal resolution, the principal limitation is posed by the available neutron flux. Even at advanced neutron sources (such as SINQ, Paul Scherrer Institut, Switzerland), the flux is limited to about 10^7^ n cm^−2 ^s^−1^. This value translates to single captured neutrons per 100 × 100μm pixel per 0.01 s acquisition time.

In order to alleviate the neutron intensity problem for the high temporal resolution imaging, we decided to utilize a neutron focussing guide. Neutron focussing guides with supermirror coatings are routinely used for number of experiments in neutron science [5], [6], however, their use for increasing the neutron flux locally within neutron images is limited [7]. This short paper presents the results of pilot tests using such experimental arrangement, thus increasing the available neutron flux at the expense of (i) the available field of view and (ii) the depreciation of the available spatial resolution.

## Test arrangement and results

A parabolic neutron guide [8] was selected due to its availability at PSI and was utilized for high-temporal neutron imaging experiments at BOA beamline [9].

BOA beamlines provide cold neutrons that possess the higher reflectivity and thus higher supermirror efficiency compared to thermal neutrons. The used neutron focussing guide had the following parameters. Its length equalled 1 m. The size of the entrance and exit windows were 25 × 25 mm × mm and 13.3 × 13.3 mm × mm, respectively. The parabolic-bent substrate is coated with a m = 3.6 supermirror.

The experimental arrangement at the BOA beamline was as follows: 40 × 40 mm × mm aperture was used. The “standard” MIDI-camera box was placed at the measuring position 2 (L = 5.0 m). The flight path has been equipped with flight tubes and beam-limiters. The focussing guide was placed upstream the detector in such a manner the focal point of the guide was positioned approximately 10 cm behind the scintillator screen (i.e. the distance between the exit window of the guide and the scintillator screen was about 300 mm). The MIDI-box was equipped with 200 μm-thick ^6^LiF/ZnS scintillators screen (from RC TRITEC, Teufen, Switzerland) and with a sCMOS detector (Hamamatsu, ORCA Flash v4.0, 6.5 μm pixel size, 2048 × 2048 pixels) coupled with a 50-mm lens (Nikon). The resulting pixel in the image equalled 56.3 μm.

**Fig. 1:**
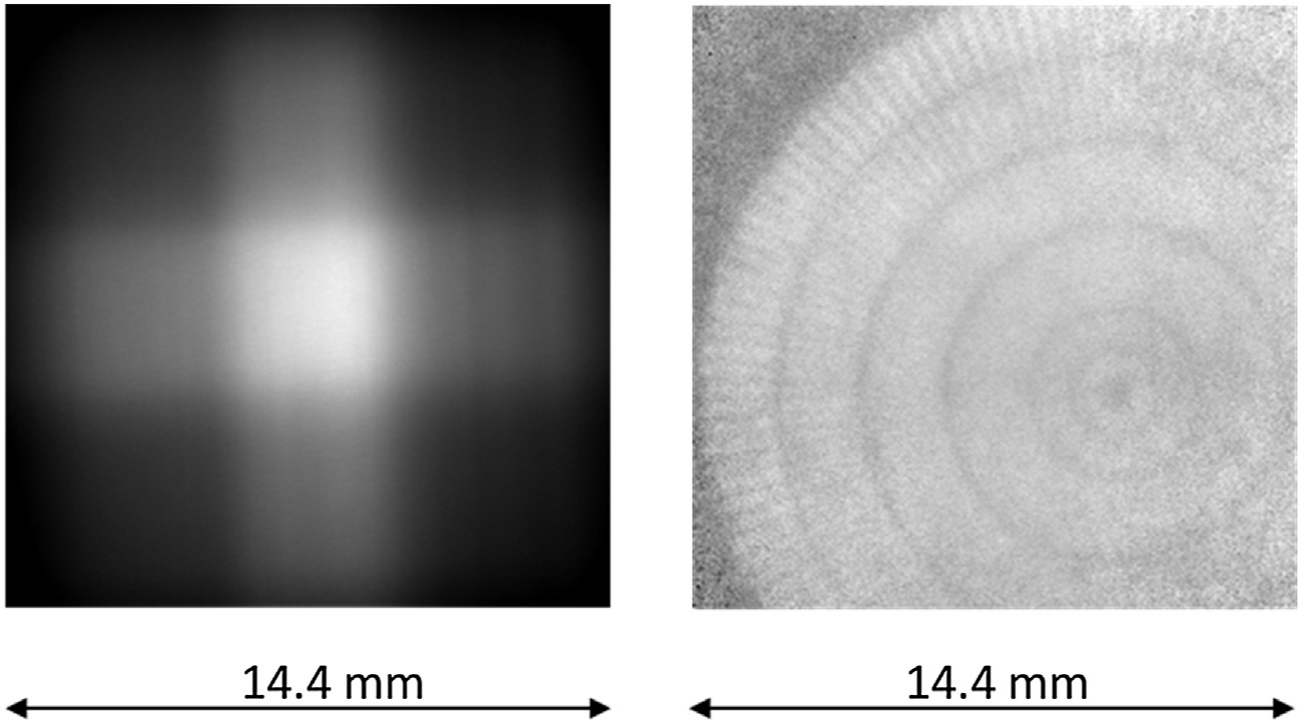
(Left) An open beam image showing the distribution of the neutron flux due to the use of the parabolic focussing guide at the BOA beamline. The centre flat-top region exhibits about order an of magnitude increase in the available flux. The image is median filtered from 100 images of 10 ms acquisition time; (right) Resolution test pattern (Gadolinium Siemens star) positioned randomly with respect to the beam profile –acquisition time as short as 10 ms.

As the neutrons were focussed into smaller area by the focussing guide, only 256 × 256 pixel area of the detector were acquired, limiting the field of view to approximately 14.4 × 14.4 mm × mm. The image showing the beam distribution in the field of view is shown in Fig. 1(left). The beam distribution exhibits clear focussing of the neutron beam both in the horizontal and in the vertical directions, creating an approximately flat-top area of about 3.5 × 3.5 mm × mm in the centre of the image. The flux at this flat-top area is approximately an order of magnitude higher than should the image be taken without the focussing guide.

Naturally, the focussing guide has a significant influence on the neutron beam divergence and thus on the spatial resolution of the resulting images. In fact, the spatial resolution in the image is clearly rather non-uniform. This is manifested in the image of the test pattern [10] that was placed about 2.5 mm from the scintillator screen shown in Fig. 1(right). From this qualitative assessment and the known thickness of the used scintillator screen, we infer that the spatial resolution throughout the entire image is not better than 200 μm even for the acquisition time of only 0.01 s.

Regarding the pilot test, we performed a model experiment that allowed us to observe the process of droplets of water (H_2_O) falling into the container (12 × 12 × 5 mm × mm × mm) filled with heavy water (D_2_O) and the subsequent process of the interaction of the two liquids. For these experiments, an in-situ titration system that allows for a remote delivery of well-defined volumes of liquids onto the sample stage was assembled. Several droplets of water (each with a volume of 0.04 ml) were dropped from a needle placed about 3 mm above the original D_2_O level in the container.

**Fig. 2:**
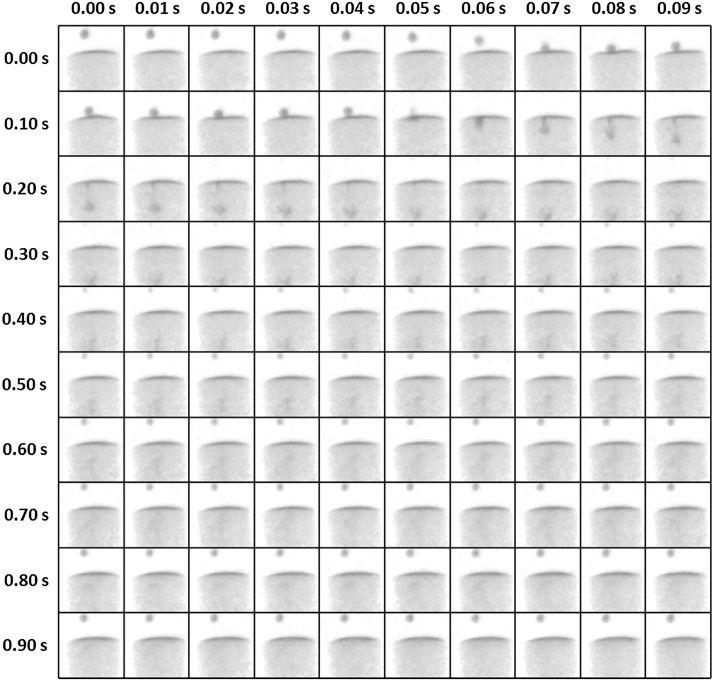
One hundred subsequent images of the high temporal resolution neutron imaging experiment showing the fall of H_2_O droplet and its subsequent mixing in the container filled with D_2_O. As the droplet is not the first one dropped into the container, the original D_2_O level is already contaminated with the H_2_O from the preceding droplets. The field of view of each image is 14.4 × 14.4 mm × mm. The images are normalized by an “open-beam” image that included the empty Teflon container allowing better visualization of the interaction of the two liquids.

**Fig. 3:**
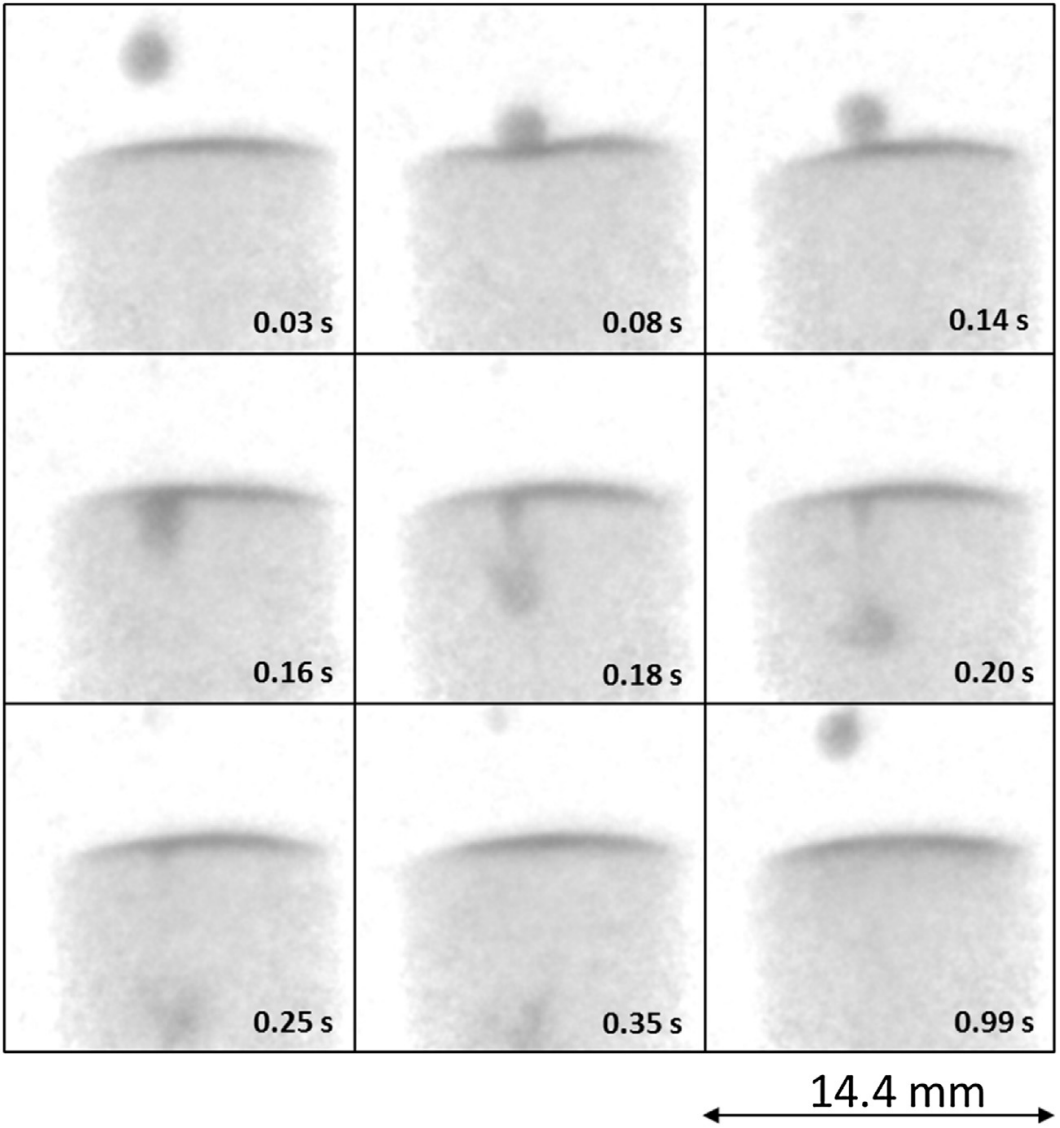
Selection of nine images from Fig. 2 showing the interaction of H_2_O droplet with D_2_O. The images are normalized by an “open-beam” image that included the empty Teflon container allowing better visualization of the interaction of the two liquids.

The acquisition time of the radiographic series was set to 10 milliseconds (i.e. 100 Hz acquisition rate). Fig. 2 shows 100 subsequent images of the fall of one such droplet into the container. As this droplet is not the first one that was dropped into the container, the original D_2_O level is already contaminated with the H_2_O from the preceding droplets. The droplet starts falling at about 0.03 s and hits the liquid surface in the container at 0.07 s. Between the times of 0.07 s and 0.14 s the droplet remains on the surface of the liquid in the container and the changes in the profile of the liquid surface in the container due to the droplet impact (in other words, the wave propagation) can be clearly observed. From the time 0.15 s the droplet starts submerging into the depth while mixing with the D_2_O volume reaching the largest depth at the time

about 0.35 s. As the density of D_2_O is higher than that of H_2_O, the H_2_O is then redistributed towards the surface of the liquid in the container by buoyancy forces, thus reinforcing the H_2_O-contaminated layer that had been formed by the previous droplets. It is noteworthy that already within app 1 s after the fall nearly all the volume of H_2_O is distributed close to the surface of the liquid (compare image at 0.03 s and 0.99 s). A selection of images showing the entire process in more detail is shown in Fig. 3.

As the longer radiographic series were actually captured, an .avi file showing the video of the extended version of this image sequence is available in the Supplementary materials of this paper.

## Discussion

From the test arrangement point of view, we must highlight again that the focussing guide has been in no way optimized for the high temporal resolution neutron imaging task. We can foresee that an optimized design (in terms of the efficient use of the available neutrons and the provision of as large as possible flat-top neutron distribution for imaging) of such guide might lead to even more favourable results in the future.

Likewise, it should be highlighted that the used scintillator screen was not optimized for the purpose of the

high-temporal resolution neutron imaging either. Two aspects of the scintillator screens should be optimized in the future for this purpose, namely, the neutron capture efficiency of the scintillator should be increased, while at the same time the light output decay time should be suppressed as much as possible.

The experiments were performed at the BOA beamline that has due to its spectrum higher sensitivity for hydrogen than that of the ICON beamline [11]. On the other hand, the ICON beamline exhibits more than two times higher flux and therefore the similar experiments are foreseen to deliver even superior results when performed at the ICON beamline or at the existing facilities of inherently higher neutron flux (e.g. ANTARES, ILL, etc.). Likewise, similar experiments are foreseen for the beamlines with already in-built neutron optics (e.g. pulse overlap diffractometer POLDI, PSI).

Regarding the presented experiments, we trust that we visualized the process of an interaction of two liquids (H_2_O and D_2_O) that are otherwise indiscernible by other probes with the very high temporal resolution of 0.01s. We foresee that rather similar experiments might provide sound experimental backing for modelling of liquid interactions [12].

The paper presents results of radiographic experiments. Naturally, this very high temporal resolution capability can be utilized for tomographic imaging with foreseen acquisition times of few seconds, thus enabling 4D investigation (3D plus time) of samples of limited size with the mentioned temporal resolution [13].

## Conclusion

This short paper presents the results of pilot tests using the combination of sCMOS detector with a parabolic neutron focussing guide. We show that such combination may provide neutron imaging of very high temporal resolution albeit at the expense of the available field of view and the depreciation of the available spatial resolution. In the pilot model experiment, we visualized the process of an interaction of two otherwise indiscernible liquids (H_2_O and D_2_O) with the temporal resolution of 0.01s. We trust that the very high temporal resolution neutron imaging capability will find further users both from the academia and the industry.
