# Supplementary figures and images for: 100 Hz neutron radiography at the BOA beamline using a parabolic focussing guide
Source: MethodsX. 2016 Oct 6;3:535–41. doi: 10.1016/j.mex.2016.10.001 (PMC5067979; doi:10.1016/j.mex.2016.10.001)

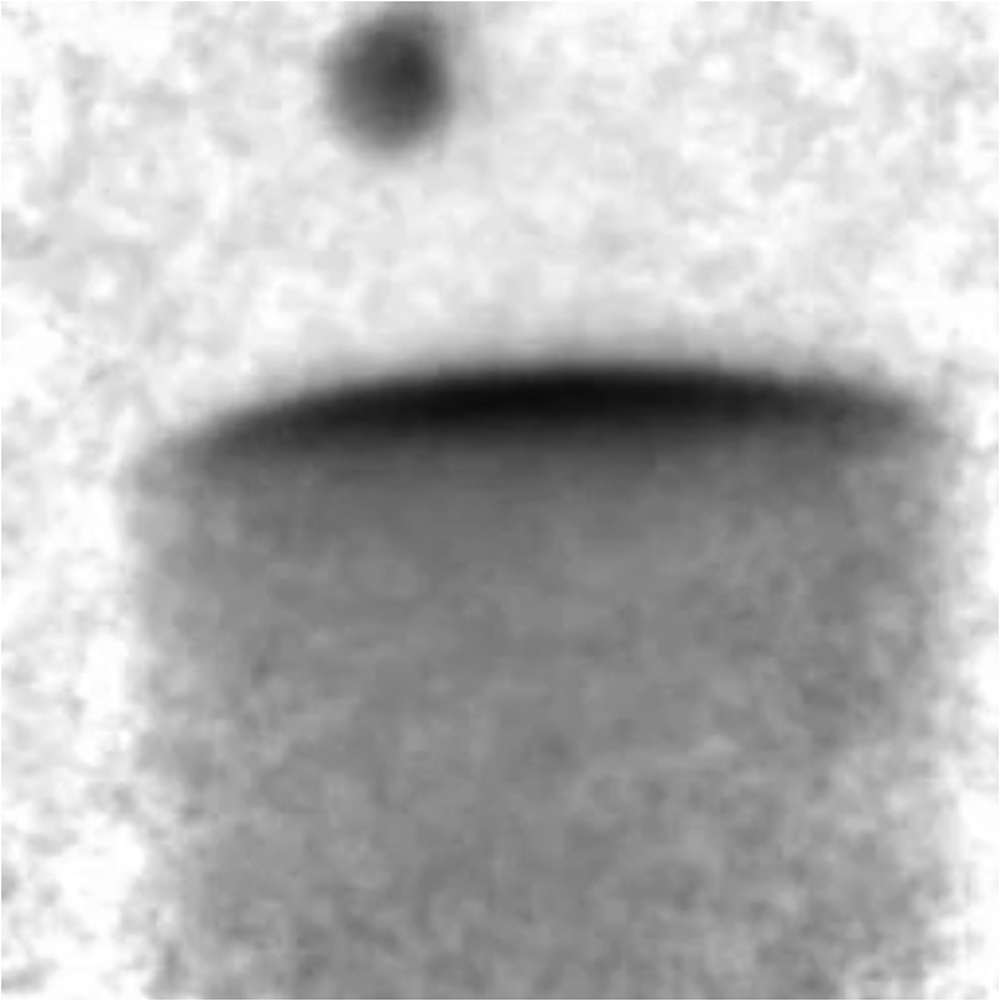

Supplement: Supplementary file 1 [file mmc1.jpg]
